# The influence of sugar–protein complexes on the thermostability of C-reactive protein (CRP)

**DOI:** 10.1038/s41598-021-92522-3

**Published:** 2021-06-21

**Authors:** Andreea Lorena Mateescu, Nicolae-Bogdan Mincu, Silvana Vasilca, Roxana Apetrei, Diana Stan, Bogdan Zorilă, Dana Stan

**Affiliations:** 1 DDS Diagnostic, 032032 Bucharest, Romania; 2 Department of Botany and Microbiology, Faculty of Biology, University of Bucharest, 060101 Bucharest, Romania; 3 Department of Life and Environmental Physics, Horia Hulubei National Institute in Physics and Nuclear Engineering, 077125 Măgurele, Romania; 4 IRASM Department – Radiation Processing Centre, Horia Hulubei” National Institute for Physics and Nuclear Engineering IFIN-HH, 30 Reactorului Street, 077125 Bucharest-Magurele, Romania; 5 Department of Analytical Chemistry, Faculty of Chemistry, University of Bucharest, Panduri Ave.# 90, 050663 Bucharest, Romania; 6 Faculty of Medicine, Titu Maiorescu University, 22 Strada Dâmbovnicului Tineretului, 040441 Bucharest, Romania

**Keywords:** Biochemistry, Biological techniques, Molecular biology, Biomarkers, Medical research

## Abstract

The purpose of the present study was to evaluate de influence of protein–sugar complexation on the stability and functionality of C-reactive protein, after exposure to constant high temperatures, in order to develop highly stable positive controls for in-vitro diagnostic tests. C-reactive protein is a plasmatic protein used as a biomarker for the diagnosis of a series of health problems such as ulcerative colitis, cardiovascular diseases, metabolic syndrome, due to its essential role in the evolution of chronic inflammation. The sugar–protein interaction was investigated using steady state and time resolved fluorescence. The results revealed that there are more than two classes of tryptophan, with different degree of accessibility for the quencher molecule. Our study also revealed that sugar–protein complexes have superior thermostability, especially after gamma irradiation at 2 kGy, the protein being stable and functional even after 22 days exposure to 40 °C.

## Introduction

**Figure 1:**
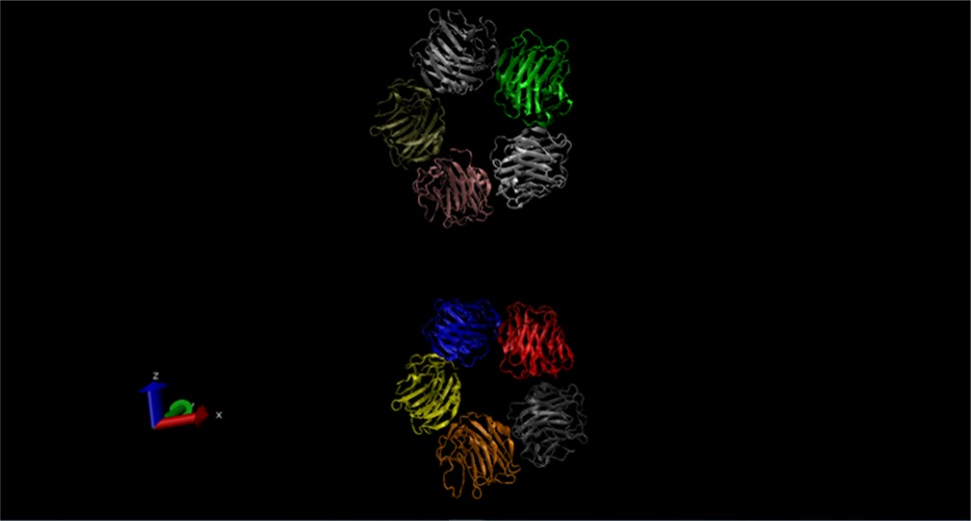
Pentameric structure of C-reactive protein obtained using the Visual Molecular Dynamics (VMD) software^2^.

C-reactive protein (CRP) is a plasmatic protein, with a pentamer structure and a molecular mass of about 110–140 kDa. CRP is a member of the pentraxin family, composed of 206 amino acids, with a pentameric, specific structure: five identical non-glycosylated globular subunits which can be clearly seen in Fig. 1^1^. High levels of CRP always indicate an inflammation in the body. The protein is produced by the organism when the blood vessels walls are inflamed and the inflammation level is directly proportional to the levels of CRP. This protein plays an essential role in the evolution of chronic inflammation, and is the result of constant deterioration of the inner walls of the arteries. These changes are the result of an unhealthy lifestyle, especially with food choices that lead to excess LDL cholesterol, triglycerides and blood sugar, as well as hypertension.

Currently, a series of rapid tests are been marketed for the detection/quantification of CRP, used as a biomarker for the assessment of various disorders or the risk of developing them. They can offer information about the active phase of autoimmune conditions such as Crohn's or ulcerative colitis, the risk of developing a cardiovascular disease and more recently studies had shown that this protein is very important in the diagnosis of the Metabolic Syndrome (MS).

The basic principle of these tests is the antigen (Ag)-antibody (Ab) interaction, which is usually very specific, but their coupling is done by Van der Waals type bonds, hydrogen, hydrophobic or ion–dipole, connections considered to be quite fragile^3^. Rapid in-vitro diagnosis kits (IVD) can sometimes offer false positive or false negative results due to cross reactivity or the phenomenon known as the "hook effect", that is why it is very important to assess the detection limit and verify them using positive controls of known concentrations. Because the targeted biomarkers are usually proteins, producing high stability positive controls for these kits is a challenge for the manufacturers.

There are a number of factors that can affect the integrity of proteins (the presence of proteases, oxidative stress, temperature, etc.), and this makes it difficult to keep proteins active. The present study aims to establish how the addition of sucrose in a CRP solution can improve the thermostability of the protein and the optimal concentration for its active maintenance, even after prolonged exposure to 40 °C ± 1 °C.

**Figure 2:**
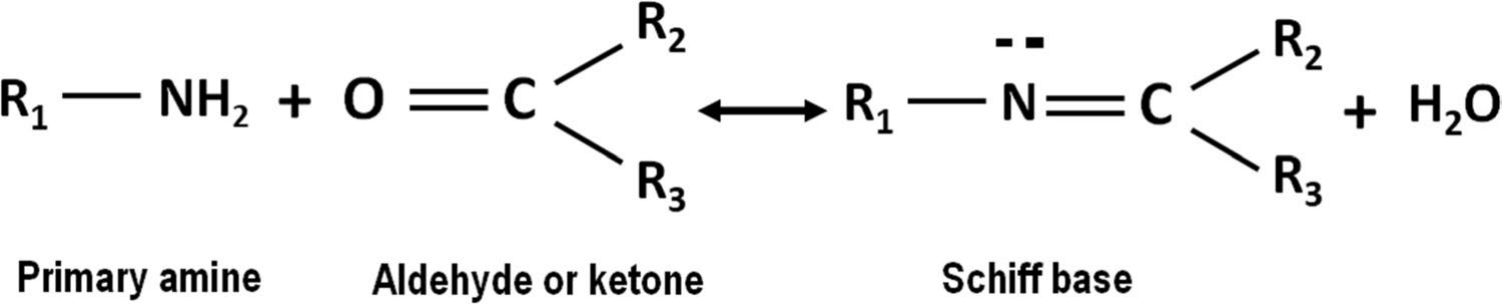
Shiff base formation.

Glycosylation is an effective method of thermal stabilization of proteins^4^. The exact mechanism of protein/sugar aggregate formation involves the interaction of an aldehyde/ketone group with an amino group in the protein, forming a structure called the Schiff’s base, followed by a series of cascading reactions leading to the formation of covalent aggregates (Fig. 2).

Data regarding the effects of sugar–protein complexation on intrinsic fluorescence show a large pallet of results. A possible explanation is that sugars are influencing the very nature of the protein-solvent interaction, with direct effect on both the tertiar and quaternar structure of the proteins^5^.

Starting from the idea of creating a highly stable CRP positive control samples for rapid detection and quantification tests for the commercial kits, we carefully analyzed the literature and encountered a few manuscripts which tested thermal stabilization of proteins in aqueous solution by the addition of sucrose, glucose or trehalose.

Rapid immunochromatographic tests for instance, do not require special storage conditions, but usually need to be maintained at room temperature. However, during the warm season, the kits can be exposed to higher temperatures during storage and transportation and all the components (including the positive control sample) should be able to maintain active after exposure to temperatures above 30 °C for at least 1 week.

Studies demonstrated that increased sugars concentrations conferred proteins increased stability to high temperature. Also, sucrose manifested a stronger stabilization effect when compared to glucose and trehalose is mainly used as a cryoprotectant^5,6^.

## Results

### Evaluation of protein functionality using the LATEX-CRP kit

 One of the first steps of the study design was determining the optimum concentration of C-reactive protein used to develop the positive control sample. Thus, the working concentrations were selected after consulting the literature regarding the interpretation of CRP values. A concentration lower than 3 mg/L is normal for a healthy adult, values between 3 and 10 mg/L mean a minor increase that can be associated with obesity, pregnancy, depression, diabetes, common cold, gingivitis, sedentary lifestyle, smoking or genetic polymorphisms, while values between 10 and 100 mg/L are associated with systemic inflammation such as rheumatoid arthritis and lupus erythematosus, or other autoimmune diseases, malignancies, myocardial infarction, pancreatitis or bronchitis. Values between 100 and 500 mg/L indicate acute or viral bacterial infections, major trauma, etc.^7^. After establishing the working concentrations for CRP, we tested them using the LATEX-CRP kit.

**Figure 3:**
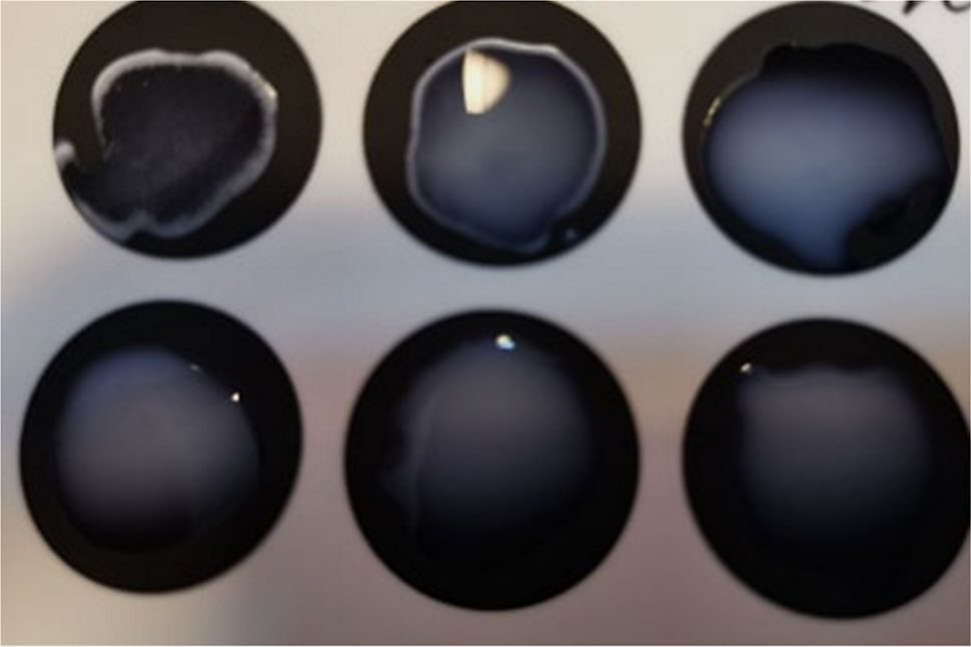
Aglutination reaction obtained using the LATEX-CRP detection kit at initial testing (T0).

The evaluation was performed at different CRP concentrations, namely: 8 mg/L; 16 mg/L and 32.5 mg/L. The samples were initially tested (T0) for verification and a strong agglutination was observed at 16 mg/L and 32.5 mg/L and its absence at 8 mg/L (Fig. 3).

The tests revealed that in the sugar free samples, the protein is functional at 40 °C for at most 48 h, at the highest concentration (32.5 mg/L), after which it suffers massive degradation and loss of immunogenicity.

Because we observed that higher concentrations positive controls samples are more stable even without the addition of sucrose or other stabilizing agents and also due to the fact that these CRP controls should also be used to evaluate the performances of semiquantitative tests, we decided to use a slightly higher concentration (65 mg/L) for the following experiments**.**

These samples, supplemented with 1 M sucrose, showed superior thermostability, the protein being intact and functional even after 20 days of incubation at 40 °C. A series of studies have shown that covalent binding of glycans to amino acid side chains of a protein can give it high thermostability^8–10^.

Some of the CRP-sucrose solutions were sent for irradiation within the National Research-Development Institute for Physics and Nuclear Engineering "Horia Hulubei" and subjected to 3 degrees of gamma irradiation: 2 kGy, 4 kGy and 6 kGy.

**Figure 4:**
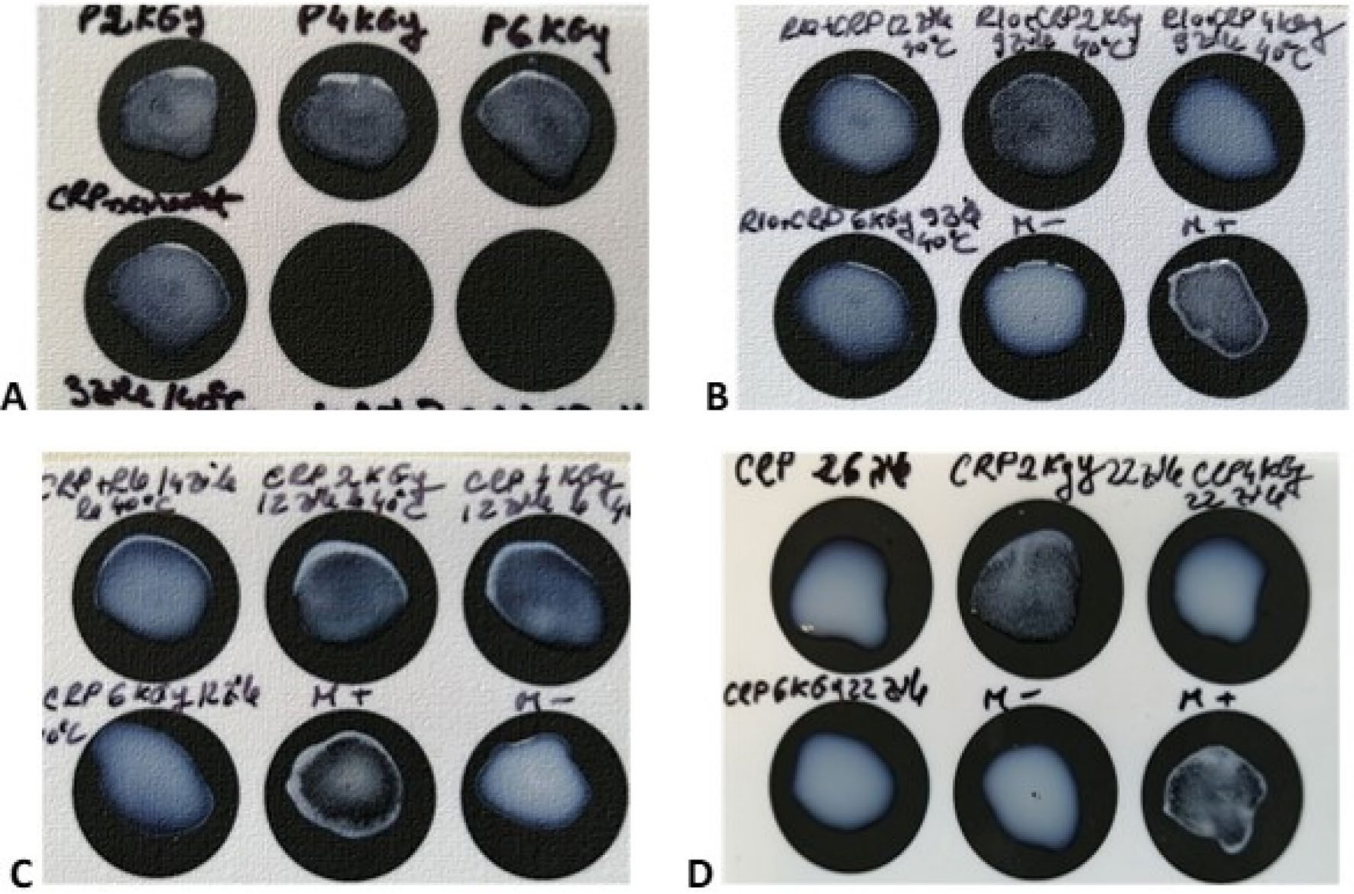
Agglutination results at different testing intervals: (**A**) after 3 days incubation at 40 °C; (**B**) after 9 days incubation at 40 °C; (**C**) after 12 days incubation at 40 °C; (**D**) after 22 days incubation at 40 °C.

Although the agglutination was weaker for the higher irradiation doses, the samples subjected to irradiation with 2 kGy proved to be even more stable than the non-irradiated ones. The tests revealed that the irradiated sugar–protein solutions were still functional and stable after 22 days incubation in stress conditions (Fig. 4).

### Fourier-transform infrared spectroscopy (FTIR)

**Figure 5:**
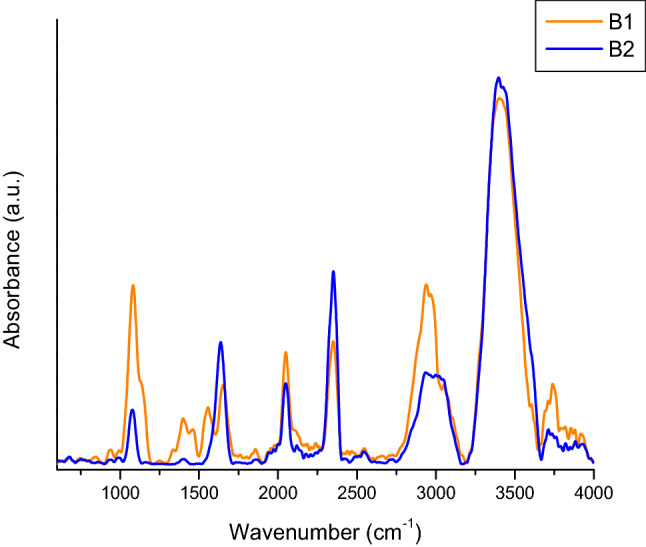
ATR-FTIR spectra for blank 1 (orange) and blank 2 (blue).

Figure 5 compares the blanks’ spectra for the two types of samples, after the spectral subtraction of water. The common bands of the two samples can be observed, such as 3387 cm^−1^ coming from the stretching vibration (ν) of the O–H group; 2363 cm^−1^ and 2334 cm^−1^ generated by the stretching vibrations of CO_2_, NO_2_; 2043 cm^−1^, from sodium azide; 1637 cm^−1^, specific for ascorbic acid and 1078 cm^−1^ from the C–O stretching vibration.

**Figure 6:**
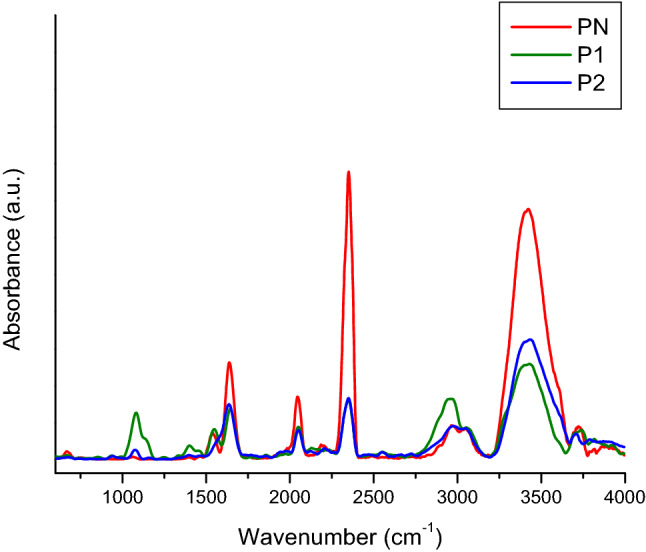
ATR-FTIR spectra of CRP standard (red), sample P1 (green), sample P2 (blue).

**Figure 7:**
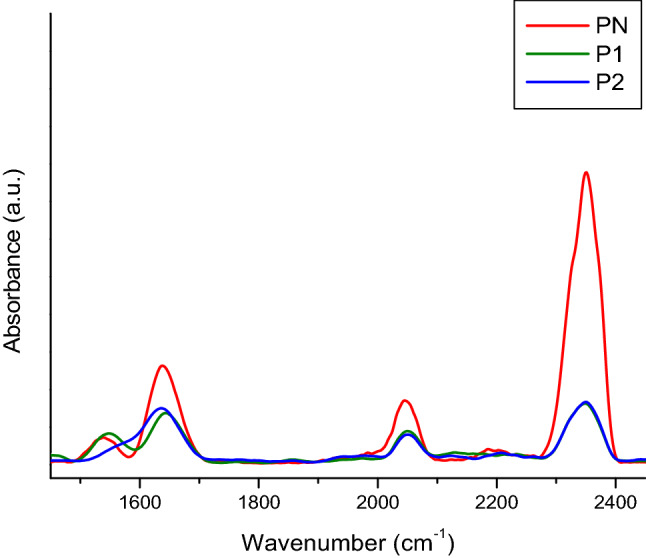
ATR-FTIR spectra of CRP standard (red), sample P1 (green), Sample P2 (blue), range from 1450 to 2450 cm^−1^.

Regarding C-reactive protein (CRP) and the positive control “Spinreact” (Figs. 6 and 7), these are similar at the molecular level, but “Spinreact” has two additional bands at 1411 cm^−1^ (δCH) and 1332 cm^−1^ (νC–C/νC–O). As for the common bands, there should be highlighted those from 1545 and 1640 cm^−1^ which are produced due to amide II, respectively amide I and the band from 2051 cm^−1^ from a thiol group νS-H. Samples’ ATR-FTIR spectra are presented after subtracting the blanks’ spectra (Fig. 5). Common bands with those of the CRP standard (2360, 2340, 1640, 1545 cm^−1^) can be observed, which attests the presence of the protein in the formulations. It is known from literature that the bands corresponding to amides I and II are the major bands in the IR spectrum of proteins. Amides I and II specific peak values correspond to those specified in the literature, namely, ~1650 cm^−1^ for amide I and ~ 1550 cm^−1^ for amide II^11,12^. In water, the bands corresponding to amide II are those around 1570–1540 cm^−1^ and include a significant contribution of the binding angle of the NH group, together with CN changes in the binding distance and other vibration-induced amide group changes, thus being extremely sensitive to the kitetics of the hydrogen atoms exchange^12^.

**Figure 8:**
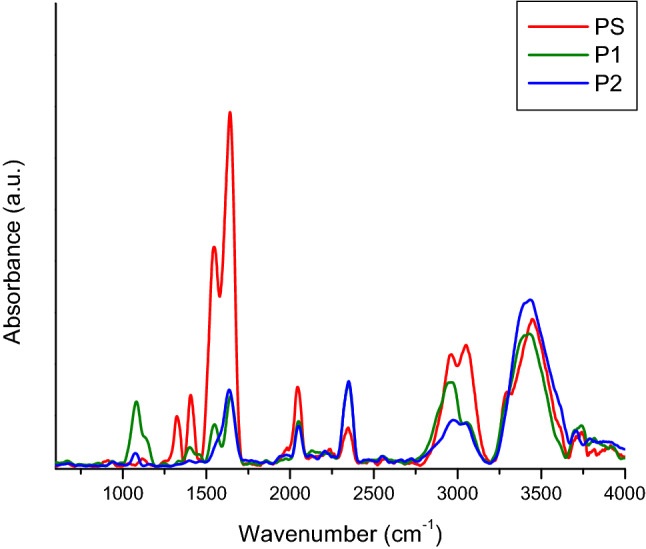
ATR-FTIR spectra for the Spinreact control (red), sample P1 (green) and sample P2 (blue).

The difference in the amide I and II specific peak amplitude between the CRP standard and the two samples (P1 and P2) reflects a lower protein concentration in the case of the two formulations. There is also a big difference between the bands related to amides I and II of the control “Spinreact” and those obtained in the case of the two formulations (Fig. 8).

### Fluorescence measurements

CRP could generate the endogenous fluorescence because it contains Tryptophan (Trp) and tyrosine (Tyr) and phenylalanine (Phe) residues. And, the fluorescence character of CRP is mainly produced by Tryptophan residue as the fluorescence intensity ratio of Trp, Tyr and Phe is 100:9:0.5^13^.

**Figure 9:**
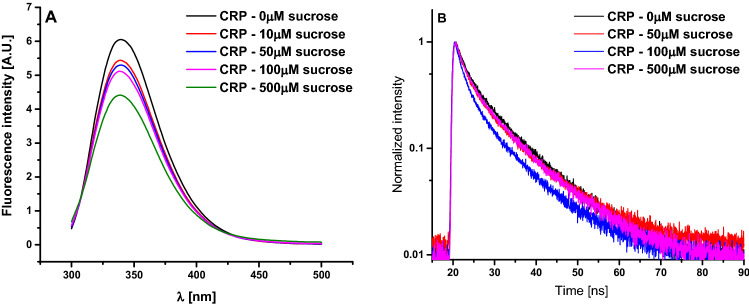
Fluorescence emission spectra for the sample containing CRP supplemented with different sucrose concentrations (**A**); Fluorescence lifetime spectra for the sample containing CRP supplemented with different sucrose concentrations (**B**).

The binding mechanism between sucrose and CRP was followed by steady-state and time resolved fluorescence. Results show that the intensity of fluorescence is significantly influenced by the concentration of sugar in the solution (Fig. 9A).

The fluorescence intensity of CRP at 339 nm gradually decreased with the addition amounts of sucrose. This revealed that sucrose could quench intrinsic fluorescence of CRP through producing a non-fluorescent complex between CRP and sucrose. Also, fluorescence lifetime slightly decreases when increasing the sucrose concentration (Fig. 9B).

**Figure 10:**
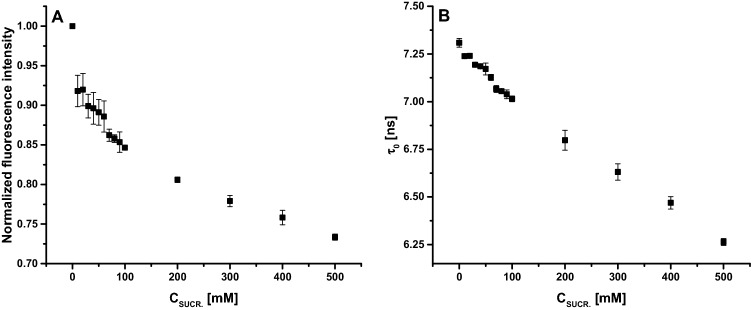
Normalized fluorescence intensity for peak maximum of emission spectra for the sample containing CRP supplemented with different sucrose concentrations (**A**); Fluorescence lifetime variation for the sample containing CRP supplemented with different sucrose concentrations (**B**).

The intensity peak of fluorescence decreases with increasing concentrations of sucrose in the environment, so that at 500 mM it reaches about 73% of the value obtained for the sample not supplemented with sucrose (Fig. 10A).

The fluorescence lifetime decreases from ≈ 7.31 ns, for CRP in buffer, with ≈ 15% in presence of 500 mM of sucrose, to approximately 6.26 ns (Fig. 10B).

To highlight possible quenching mechanisms that occur in the interaction between CRP and sucrose, the fluorescence quenching experiments were carried out, at 25 °C, and an attempt was made to obtain the quenching constant from Stern–Volmer equation^14–16^:

$$\frac{{F_{0} }}{F} = \frac{{\tau _{0} }}{\tau } = 1 + K_{{SV}} \left[ Q \right] = 1 + k_{b} \cdot \tau _{0} \left[ Q \right]$$

where *F* and *F*_*0*_ are the fluorescence intensities with or without sucrose, respectively, *τ* and *τ*_*0*_ are the mean fluorescence lifetimes of Tryptophan residues, from CRP structure, with or without sucrose, [*Q*] is the concentration of sucrose, *K*_*SV*_ is the quenching constant (Stern–Volmer constant), *k*_*b*_ is the quenching rate constant of protein (bimolecular constant) and *τ*_*0*_ is the average fluorescence lifetime of CRP without sucrose and its value is ≈ 7.31 ns, experimentally determined.

**Figure 11:**
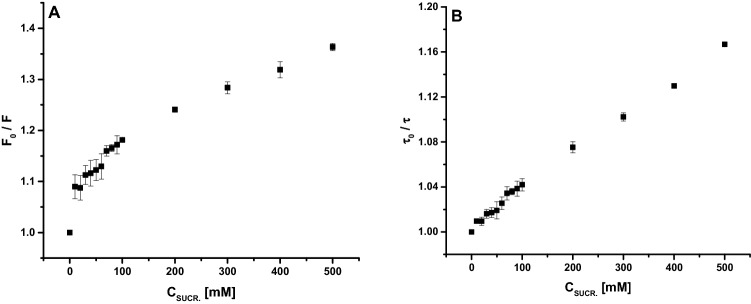
Stern–Volmer plots of CRP in presence of increasing concentrations of sucrose: steady state fluorescence (**A**) and fluorescence lifetime (**B**).

In general, two types of fluorescence quenching can be distinguished: dynamic quenching and static quenching. The static and dynamic quenching can be differentiated by analysis of Stern–Volmer plots described by previous equation and, in our case, represented in Fig. 11A for steady state fluorescence and Fig. 11B for fluorescence lifetime.

When only dynamic quenching or static quenching occur, the Stern–Volmer plots of steady state fluorescence data should be a straight line with a slope between 0 and 1. Static quenching can be distinguished from dynamic quenching using Stern–Volmer plot of fluorescence lifetimes, in that case (static quenching) the slope of graph being equal to 0^15^.

In our case, the quenching of Tryptophan, from CRP primary structure, by sucrose presented a pronounced downward curvature, this implying two or more classes of fluorophores with different degrees of accessibility for quencher molecules (Fig. 3A). We tested, using the obtained data, the method of analysis of fluorescence quenching using the modified Stern–Volmer plots (data not shown)^15^, plotting the variation of (*F*_0_/*F*_0_ – *F*) dependence by (1/[*Q*]), but also within this analysis method the obtained graph showed a downward curvature. This implies that there are more than two classes of tryptophan molecules with different degrees of accessibility for sucrose, CRP protein having in his primary structure six Tryptophan residues^17^.

Our results are in opposition to those obtained in another study, in which glucose and glycerol were added to samples with human glucokinase, and glucose binding seems to determine an increase of the fluorescence quantum yield, of almost 2 times^18^. This may be due to factors such as protein type, amino acid composition, but also selected sugar. On the other hand, other researchers studying the interaction between human serum albumin (HAS) and 2-amino-6-hydroxy-4-(4-N,N-dimethylaminophenyl)-pyrimidine-5-carbonitrile (AHDMAPPC), have reported that the fluorescence intensity reduced gradually, as the concentration of AHDMAPPC got more and more higher in the sample. They concluded that the effect of extinguishing fluorescence was due to the formation of a non-fluorescent complex^19^.

Other studies have also reported that high concentrations of sugars such us sucrose and glucose can increase thermostability, also mentioning that a stronger stabilization effect was observed in sucrose samples^6^.

**Figure 12:**
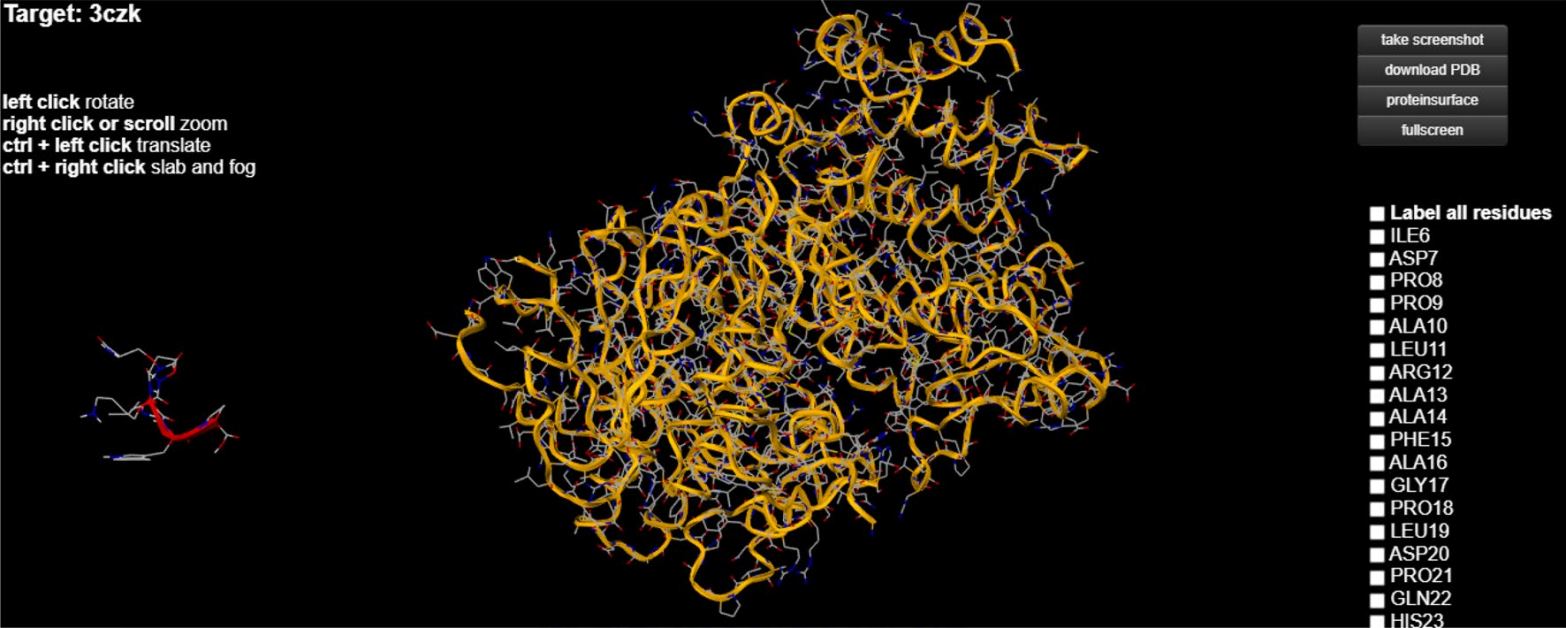
Structures of the two test molecules (C-reactive protein and sucrose) obtained using 1-CLICK DOCKING Software from the mcule platform, version from 2011, https://mcule.com/apps/1-click-docking/.

In an attempt to establish the molecular mechanism by which fluorescence is extinguished, we used the 1-click docking software from “mcule”. Both molecules were loaded in the database (Fig. 12) and after selecting the proper binding center, a series of docking experiments were done.

**Figure 13:**
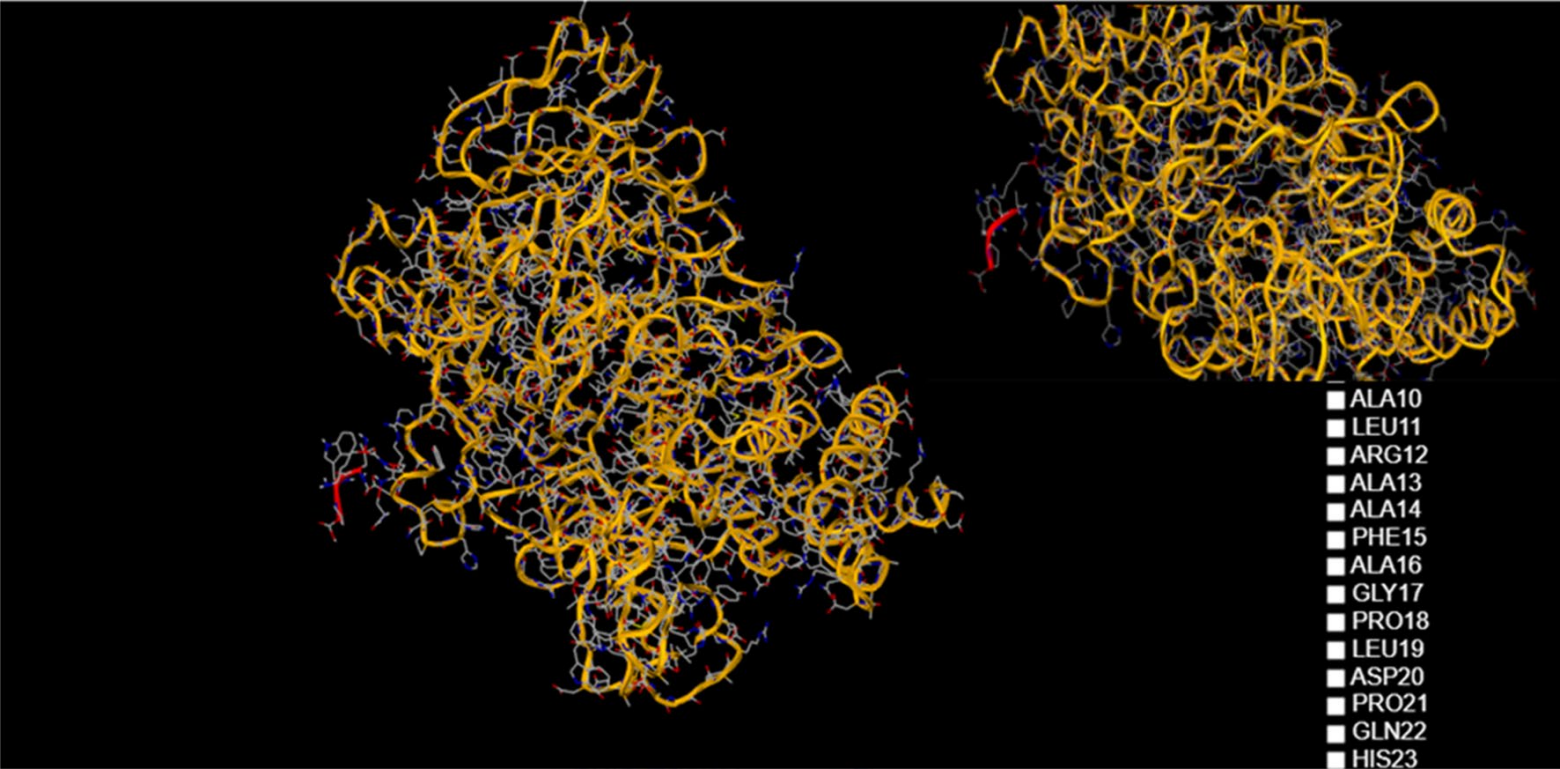
Result of the first docking experiment, with a score of -3.21 obtained using 1-CLICK DOCKING Software from the mcule platform, version from 2011, https://mcule.com/apps/1-click-docking/.

**Figure 14:**
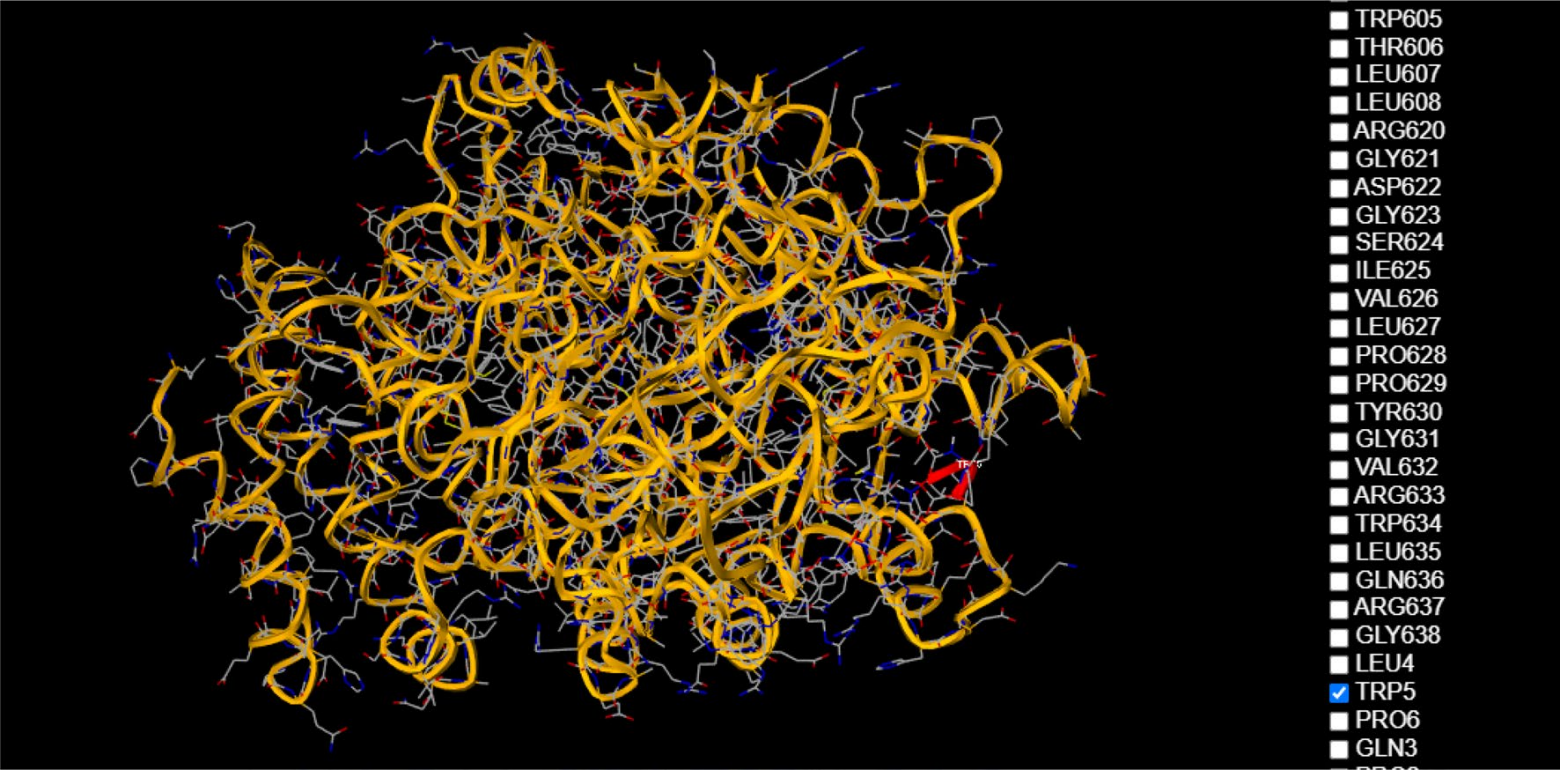
Bounding of the sucrose molecule near TRP5 obtained using 1-CLICK DOCKING Software from the mcule platform, version from 2011, https://mcule.com/apps/1-click-docking/.

The first docking had a score of − 3.2 (Fig. 13) and the second one − 4.9 (Fig. 14) and revealed that sucrose was bound near TRP5. The more negative the docking score is, the better the match. Since there are a number of tryptophans located on the outside of the molecule (TRP187, TRP524, TRP605, etc.) these simulations suggest that extinguishing fluorescence with increasing sucrose concentration may be correlated with blocking the intrinsic fluorescence of tryptophan and perhaps other fluorescent aminoacids such as tyrosine and phenylalanine.

Compared to TYR and PHE, TRP is the most abundant and complex, exhibitting high sensitivity to the environment and more than two different fluorescent lifetimes^20^. Studies show that proteins containing one TRP undergo multiexponential fluoresence decay. A hypothesis for this decay is the existence of different rotameric conformations of the aminoacid side chain^21^.

Although we selected a series of binding centers located near TRP, the best fit appeared to be near the TRP5 region.

## Discussion

Our results confirmed the data provided by the literature regarding the thermal stabilization of proteins by complexing them with sugars. The ATR-FTIR analysis revealed that the protein has not suffered any structural damage, after exposure to 40 °C, as demonstrated by the presence of the main characteristic picks for CRP: carbonyl stretching vibration (amide I), nitrogen–hydrogen deformation vibration (amide II) and N–H stretching vibration. However, the loss of immunogenicity was observed when performing the LATEX CRP test, which could be due to some conformational changes.

The interaction between sucrose and CRP was highlighted using steady state and time resolved fluorescence. From the steady state measurements, it resulted that there are more than two classes of Tryptophan with different degrees of accessibility for sucrose. From combination of steady state and time resolved fluorescence measurements resulted that both types of quenching occur, but further studies should be considered, especially in the field of small molar ratios, in our case for concentrations of sucrose up to 10 mM. The fluorescence studies also revealed that by adding increasing concentrations of sucrose the solution fluorescence gradually decreased, until reaching a plateau level. We concluded that this plateau level could correspond to the optimum sucrose concentration in order to achieve complete complexation of protein molecules with the sugar.

The molecular docking experiment revealed that sucrose was bound near TRP5, this suggests that fluorescence extinction could be caused by blocking the intrinsic fluorescence of TRP, or other amino acids such as TYR and PHE.

Samples prepared using the optimum ratio between protein and sucrose concentration remained active even after 22 days exposure to 40 °C, ± 1 °C. Based on these findings, highly stable positive controls for rapid qualitative and quantitative testing of CRP can be developed, with reduced costs and maximum efficiency.

The present study helps manufacturers of rapid “in vitro” diagnostic tests to produce kits with high performance and stability. The introduction of positive controls samples in the rapid kits for the detection/quantification of the CRP protein, not only helps determine the test performance, but also avoid erroneous results. Also, as previously mentioned, data regarding the effects of sugar–protein complexation on intrinsic fluorescence are often contradictory and this suggests that not all sugar–protein interactions are identical, but the reason behind this observation is yet to be determined. The current study also revealed that the exposure of the CRP-sucrose solutions to Gamma radiation can help improve their stability at high temperatures, especially after subjecting them to a dose of 2 kGy, which was not previously described in the literature according to our knowledge.

## Methods

Samples were prepared with identical CRP concentration (65 mg/L), with and without the addition of sucrose. Sodium azide was added as a preservative and ascorbic acid to protect the protein from reactive oxygen species. C-reactive protein was provided by My BioSource, and sucrose by Sigma-Aldrich. The CRP solutions were prepared using phosphate buffered saline, supplied by Sigma-Aldrich. Both sets of samples (with and without sucrose) were incubated at 40 °C ± 1 °C and tests were made periodically for the assessment of the integrity and functionality of the protein.

### Evaluation of protein functionality using the LATEX-CRP kit

The kit was supplied by DDS Diagnostic and it was designed for the detection of the C-reactive protein. LATEX-CRP is an agglutination based rapid test for the qualitative and semiquantitative detection of CRP, which is based on latex particles bound to specific antibodies against human C-reactive protein. Sample testing was performed according to the steps below:

- 40 µL of sample, positive control and negative control were added into separate circles of the test card
- 40 µL of latex solution was added to each circle of the card, after carefully shaking the bottle for resuspension of the latex particles
- both drops (sample and latex) were mixed
- the card was rotated at 100 rpm for 2 min.

### Testing by vibrational spectroscopy (Fourier-transform infrared spectroscopy)

An attempt to establish changes induced by subjecting the protein to accelerated aging conditions was performed using FTIR (Fourier-transform infrared spectroscopy). In this study, the samples examined were as follows: P1—sucrose free sample; B1—blank buffer for P1; P2—sucrose free sample incubated at 40 °C, for 5 days; B2—blank buffer P2; PN—positive control supplied by Hytest (concentration 2.2 mg/mL); PS – positive control supplied by SPINREACT (concentration > 20 mg/L).

The spectrometer used for the analysis was FT-IR Vertex 70, equipped with Raman RAM II module and IR probe module for non-destructive analysis. Data acquisition of ATR-FTIR spectra was performed in reflection on the 600–4000 cm^−1^ spectral domain, with a resolution of 4 cm^−1^ and 256 scans per sample. For data interpretation, the obtained spectra were processed with OPUS software ver. 6.5 (produced by Bruker Optics, Germany) and the represented spectra are the average of at least 5 measurements performed for each sample.

### Fluorescence measurements

For fluorescence experiments CRP was used at a final concentration of 13 µg/mL. Sucrose dissolved in TRIS buffer (pH 8.5) was used as quencher of Tryptophan fluorescence. The quenching of CRP fluorescence was carried out by successive additions of the quencher in the protein solution (steps of 10 mM—for the range from 0 to 100 mM and 100 mM steps between 100 and 500 mM). The overall dilution did not exceed 5.0%. The solutions were mixed using magnetic stirring and kept 7 min before measurements. The binding mechanism was followed at 25 °C.

### Fluorescence spectroscopy

Steady-state fluorescence measurements were performed using a FluoroMax 3 spectrofluorometer (Horiba Jobin Yvon, Edison, NJ, USA) equipped with a Peltier thermostat cell holder and magnetic stirrer. The excitation wavelength was 280 nm and emission spectra were recorded in the spectral range 300–500 nm, with 1 nm step. The slit for the excitation and emission monochromator was 3 nm. The spectra recorded were first corrected for the spectral sensitivity of the emission channel of the spectrofluorometer. A second correction for Raman and scattering artifacts was done by subtracting from the spectra the contribution of the buffer solution. All records were made at 25 °C.

### Time resolved fluorescence spectroscopy

Fluorescence lifetime measurements were performed using a home-made setup for time-resolved fluorescence measurements, based on time-correlated single-photon counting technique. The excitation light source was a sub-nanosecond pulsed LED head PLS 280 (280 nm, spectral width < 10 nm, pulse width 600 ps) controlled with the PDL 800-D driver, both from PicoQuant (Berlin, Germany). The decay curves were recorded using the Time-Correlated Single Photon Counting module TimeHarp 200 and a photomultiplier detector, PMA182-P-M also from PicoQuant. The fluorescence decays were collected using a long pass filter, UV 325 nm (Chroma Technologies, Bellows Falls, VT, USA).

The instrument response function (IRF) was obtained through the use of a scattering Ludox solution. During the measurements, the cuvette temperature was kept at 25 °C using a Peltier thermostat and continuously stirred using a magnetic stirrer. Fluorescence decay data were deconvoluted using the FluoFit software package from PicoQuant. The number of counts found in the peak of the curves was set to 5000, before the running the measurement protocol. The quality of the fit was judged by the reduced χ^2^ value, which takes a value around 1 or slightly larger for a high-quality fit.

The average fluorescence lifetime ($\bar{\tau }$) was calculated using the following Eq. (1):

1 $$\bar{\tau } = \frac{{\sum \alpha _{i} \tau _{i}^{2} }}{{\sum \alpha _{i} \tau _{i} }}$$

where, *α*_*i*_ represents the normalized amplitude corresponding to the ith decay time constant, *τ*_*i*_.
